# Epigallocatechin-3-Gallate Protects Pro-Acinar Epithelia Against Salivary Gland Radiation Injury

**DOI:** 10.3390/ijms22063162

**Published:** 2021-03-19

**Authors:** Erni Sulistiyani, James M. Brimson, Ajjima Chansaenroj, Ladawan Sariya, Ganokon Urkasemsin, Sornjarod Oonsiri, Tewin Tencomnao, Anjalee Vacharaksa, Risa Chaisuparat, Joao N. Ferreira

**Affiliations:** 1 Exocrine Gland Biology and Regeneration Research Group, Faculty of Dentistry, Chulalongkorn University, Bangkok 10330, Thailand; 6175855432@student.chula.ac.th (E.S.); a.chansaenroj@gmail.com (A.C.); Risa.C@chula.ac.th (R.C.); 2 Natural Products for Neuroprotection and Anti-Ageing Research Unit, Department of Clinical Chemistry, Faculty of Allied Health Sciences, Chulalongkorn University, Bangkok 10330, Thailand; james.b@chula.ac.th (J.M.B.); tewin.t@chula.ac.th (T.T.); 3 The Monitoring and Surveillance Center for Zoonotic Diseases in Wildlife and Exotic Animals, Faculty of Veterinary Science, Mahidol University, Nakhon Pathom 73170, Thailand; ladawan.sar@mahidol.edu; 4 Department of Preclinical and Applied Animal Science, Faculty of Veterinary Science, Mahidol University, Nakhon Pathom 73170, Thailand; ganokon.urk@mahidol.edu; 5 Division of Radiation Oncology, Department of Radiology, King Chulalongkorn Memorial Hospital, Bangkok 10330, Thailand; nuunon@yahoo.com; 6 Department of Microbiology, Faculty of Dentistry, Chulalongkorn University, Bangkok 10330, Thailand; Anjalee.V@chula.ac.th; 7 Department of Oral Pathology, Faculty of Dentistry, Chulalongkorn University, Bangkok 10330, Thailand; 8 Faculty of Dentistry, National University of Singapore, Singapore 119077, Singapore

**Keywords:** salivary glands, hyposalivation, radiotherapy, epigallocatechin-3-gallate

## Abstract

Antioxidant agents are promising pharmaceuticals to prevent salivary gland (SG) epithelial injury from radiotherapy and their associated irreversible dry mouth symptoms. Epigallocatechin-3-gallate (EGCG) is a well-known antioxidant that can exert growth or inhibitory biological effects in normal or pathological tissues leading to disease prevention. The effects of EGCG in the various SG epithelial compartments are poorly understood during homeostasis and upon radiation (IR) injury. This study aims to: (1) determine whether EGCG can support epithelial proliferation during homeostasis; and (2) investigate what epithelial cells are protected by EGCG from IR injury. Ex vivo mouse SG were treated with EGCG from 7.5–30 µg/mL for up to 72 h. Next, SG epithelial branching morphogenesis was evaluated by bright-field microscopy, immunofluorescence, and gene expression arrays. To establish IR injury models, linear accelerator (LINAC) technologies were utilized, and radiation doses optimized. EGCG epithelial effects in these injury models were assessed using light, confocal and electron microscopy, the Griess assay, immunohistochemistry, and gene arrays. SG pretreated with EGCG 7.5 µg/mL promoted epithelial proliferation and the development of pro-acinar buds and ducts in regular homeostasis. Furthermore, EGCG increased the populations of epithelial progenitors in buds and ducts and pro-acinar cells, most probably due to its observed antioxidant activity after IR injury, which prevented epithelial apoptosis. Future studies will assess the potential for nanocarriers to increase the oral bioavailability of EGCG.

## 1. Introduction

The Global Cancer Observatory from the World Health Organization estimates that head and neck cancer (HNC) incidence will reach approximately 1.5 million cases worldwide in 2020 [1]. Nevertheless, radiotherapy (RT) remains one of the cornerstone standard therapies to attenuate HNC progression [2,3]. The advancement of linear accelerator (LINAC) technologies together with intensity-modulated radiation therapy (IMRT) techniques have enhanced the precision and efficiency of fractionated RT for HNC [2,3,4]. Emerging research efforts have also been undertaken to understand these RT technologies’ ability to spare the function of neighboring healthy tissues or organs like the salivary glands (SG) [5,6,7,8].

Despite these research advances, a large majority of HNC patients who undergo RT display irreversible dry mouth symptoms (xerostomia) due to high radiation sensitivity of salivary gland (SG) secretory cells [5,8,9]. This gland damage is thought to be triggered by an RT-induced loss of acinar cells and a potential impairment of the parasympathetic innervation and vascularization [10,11,12,13]. Hence, the remaining integral SG stem/progenitor cells post-RT will define the true regenerative ability of the SG organ.

Cytoprotectant agents like amifostine have been recommended to prevent RT damage to SG cells [6,14]. Amifostine is the only US Food and Drug Administration (FDA) approved drug for this prevention strategy [15]. In Phase III clinical trials, amifostine was found to reduce xerostomia severity in subjects with grade two and above; however, more than 50% of subjects still presented acute xerostomia symptoms and oral mucosa inflammation [16,17]. Moreover, amifostine has a very narrow therapeutic window [14]. Therefore, frequent administration is required leading to severe side effects in more than half of the treated individuals [17,18]. These side effects can lead to the discontinuation of amifostine treatment and RT delay in 25% of HNC patients [18]. The high frequency of reported side effects and its high cost and low-quality evidence of efficacy from several clinical trials make amifostine a less promising pharmacological approach [14]. Thus, novel pharmaceuticals are necessary to prevent SG damage and maintain the acinar epithelial and stem/progenitor cell populations in the SG organ.

Antioxidant compounds can be suitable alternatives to protect non-cancer cells from RT damage [19]. Epigallocatechin-3-gallate (EGCG) is a well-known antioxidant and cytoprotective agent. It is a major flavonoid generated from the process of heating *Camellia sinensis* (green tea) leaves [20,21,22,23]. EGCG can exert cytoprotective effects in normal epithelial cells/tissues in vitro [24,25] and in clinical trials [20]. Moreover, EGCG at 200 mg per day has a remarkable safety profile with limited side effects [20,22].

For example, to maintain homeostasis in the kidney, EGCG induced antioxidant activity and cytoprotection in mouse tubular epithelial cells after exposure to reactive oxygen species [25]. In the context of exocrine glands, EGCG treatment to human breast epithelial cells also promoted cytoprotection [26,27]. However, EGCG has not been thoroughly investigated in salivary exocrine glands during normal physiological conditions and upon injury. Investigations have only been performed in immortalized acinar epithelial SG cell cultures that were genetically modified *in vitro*; however, such reports have shown promising outcomes favoring high viability rates (~100%) with EGCG concentrations ranging from 5.75 to 23 µg/mL (12.5–50 µM) in normal physiological conditions [28]. Conversely, in the same study, EGCG at 23 µg/mL decreased viability in ductal epithelial cells. No comprehensive studies have been conducted to determine the biological effects of EGCG in both pro-acinar and ductal epithelial compartments of the intact SG organ, and most importantly on whether putative epithelial stem/progenitor cell populations from those compartments are well preserved after radiation injury induced by advanced LINAC technologies. These studies would be essential to improve SG regeneration, restore salivation, and enhance the quality of life of HNC patients [8,14,29].

Altogether, this study aims to: (1) determine whether EGCG can support pro-acinar epithelial proliferation and maturation during SG homeostasis; and (2) investigate how EGCG can protect the epithelial cell populations and their putative progenitors from radiation injury induced by LINAC.

## 2. Results

**Figure 1:**
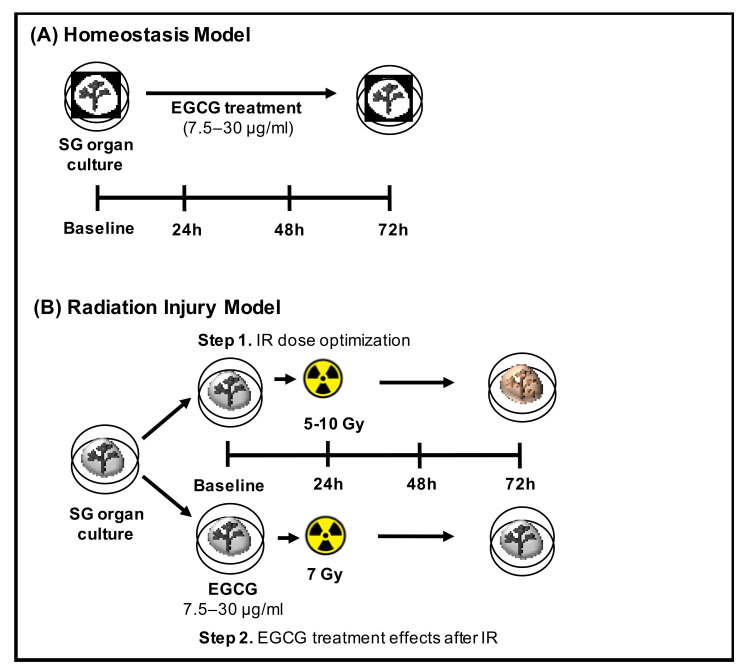
SG epithelial homeostasis and radiation injury models. (**A**) SG organ culture with EGCG treatment during homeostasis. (**B**) SG organ culture with elicited irradiation (IR) injury and EGCG treatment.

The two ex vivo SG experimental models used in this study are displayed in Figure 1. SG epithelial homeostasis models were utilized first for optimizing EGCG concentrations that support epithelial growth and maturation (Figure 1A), and SG epithelial injury models induced by LINAC radiation were performed thereafter with the optimal range of EGCG concentrations (Figure 1B).

### 2.1. SG Epithelial Homeostasis

**Figure 2:**
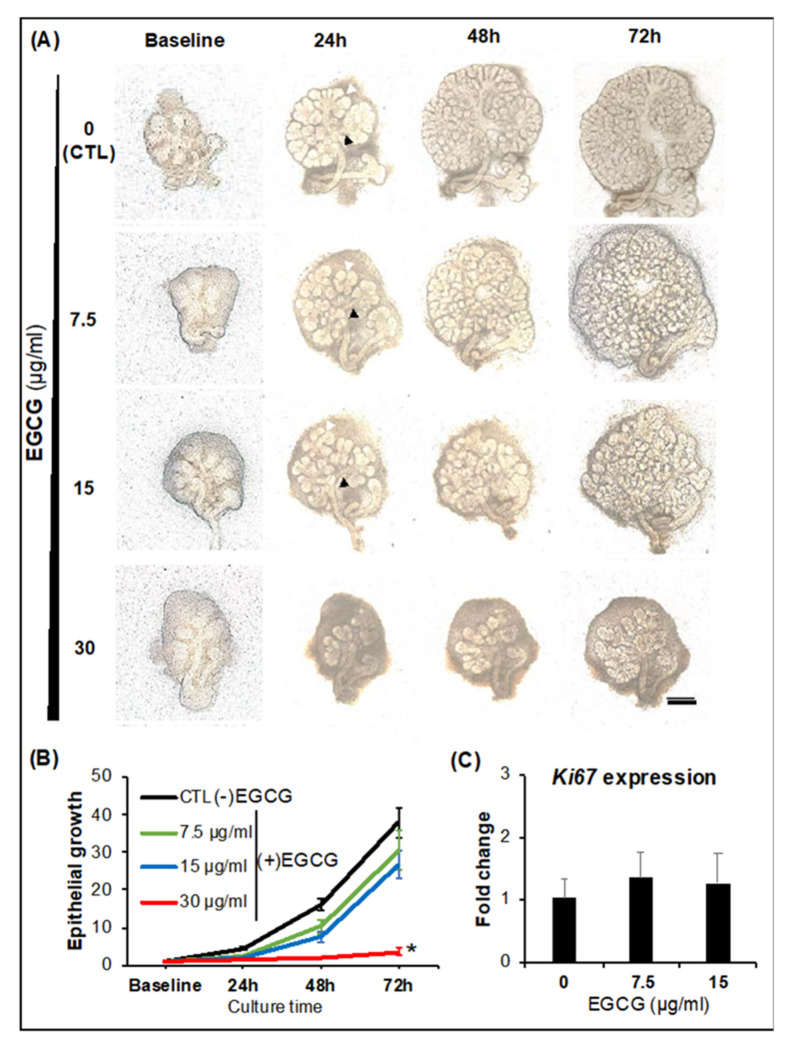
EGCG at 7.5–15μg/mL supported SG epithelial growth and proliferation during homeostasis. (**A**) Bright-field microscopy micrographs of SG cultured with EGCG for 72 h. Black arrowheads represent secondary duct formation. White arrowheads indicate epithelial bud clefting. Mag.: 4×. Scale bar: 200 μm. (**B**) Quantification of SG epithelial growth when SG exposed to different EGCG concentrations. Error bars represent SEM from *n* = 10–12. * *p* < 0.0001 when compared to control by one-way ANOVA with Dunnett’s *post hoc* analysis. (**C**) Proliferation activity upon measuring the expression of a mitotic marker at 72 h. *Y*-axis represents the fold change of *Mki67* gene relative to baseline levels and normalized to *Rsp29* (housekeeping gene). Error bars represent SEM from *n* = 3. No statistically significant differences by one-way ANOVA with Tukey’s *post hoc* analysis.

After 72 h of culture, the epithelial end bud outgrowth of the SG exponentially increased over 26-fold in EGCG treatment groups ranging from 7.5 to 15 µg/mL (Figure 2A,B), as well as the formation of epithelial clefts (white arrowheads in Figure 2A) and secondary epithelial ducts (black arrowheads in Figure 2A). These outcomes were not significantly different from the positive controls ((−) EGCG or untreated). EGCG at 30 µg/mL delayed the epithelial cleft and secondary duct formation from 24 h onwards in Figure 2A. Consequently, EGCG at the same concentration also inhibited the exponential epithelial bud outgrowth during 72 h as per the flattened curve seen in Figure 2B (*p* < 0.0001). EGCG 7.5–15 µg/mL treated glands followed a regular epithelial growth pattern that is exponential in nature as these developing glands undergo a high rate of epithelial proliferation and turnover while branching morphogenesis occurs as observed in untreated glands in Figure 2B [13,30]. To further confirm this elevated epithelial proliferation rate, EGCG-treated glands (7.5 and 15 µg/mL) were checked in gene arrays and proteomic immunofluorescence studies for other proliferation markers. In Figure 2C, as expected, these EGCG-treated glands supported the expression of Ki67 mitotic marker (*Mki67* gene) like untreated controls. This indicates that EGCG at 7.5 and 15 µg/mL can promote epithelial branching morphogenesis with regular cleft and duct formation, epithelial proliferation, and turnover. As previously reported by Yamamoto and colleagues [28], this range of EGCG concentrations also supported immortalized SG cell lines’ proliferation.

**Figure 3:**
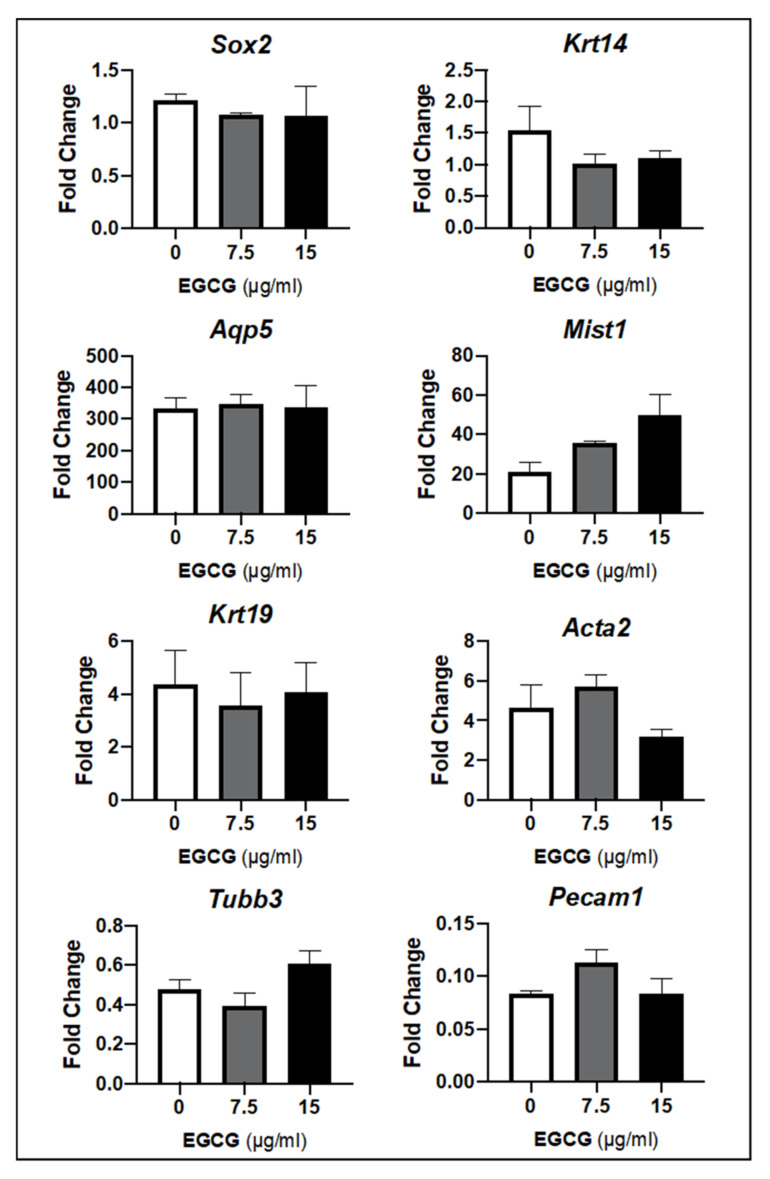
Expression of SG stem/progenitor cell, acinar and ductal epithelial, myoepithelial, neuronal, and vascular markers in the SG after EGCG treatment at low (7.5μg/mL) and high (15μg/mL) concentrations remain comparable. *Y*-axis represents fold change relative to baseline levels and normalized to *Rsp29* (housekeeping gene). Error bars represent SEM from *n* = 3, and each triplicate contains the RNA lysates of 3–4 glands. No statistically significant differences by one-way ANOVA with Tukey’s *post hoc* analysis. *Sox2:* SRY (sex-determining region Y)-box 2; *Krt14*: Cytokeratin 14; *Aqp5*: Aquaporin 5; *Mist1*: Class A basic helix-loop-helix protein 15; *Krt19*: Cytokeratin 19; *Acta2*: actin alpha 2, smooth muscle; *Tubb3*: Tubulin Beta 3 Class III; *Pecam1*: Platelet and Endothelial Cell Adhesion Molecule 1.

In Figure 3, gene expression arrays in untreated and treated SMG with 7.5 and 15 µg/mL of EGCG showed similar expression profiles with different SG-specific markers. The expression of *Sox2* and cytokeratin 14 (*Krt14*), which are known SG epithelial stem/progenitors, did not suffer any significant variations from baseline to 72 h. Regarding acinar epithelial markers such as *Aqp5* and *Mist1*, there was a remarkable upregulation across all experimental groups with no significant differences upon multiple comparisons between groups. However, when EGCG concentration increased to 15 µg/mL, the mean fold change of *Mist1* expression also increased relative to the 7.5 µg/mL EGCG (Figure 3). Thus, this suggested that EGCG can maintain the epithelial progenitors and enrich the pro-acinar epithelial compartment in the homeostasis SG model.

As for SG ductal and myoepithelial markers in Figure 3, such as *Krt19* and *Acta2*, respectively, no relevant differences in fold change were observed between untreated and EGCG-treated glands, although a 3- to 5-fold upregulation from baseline was noted. Additional acinar and ductal epithelial genetic markers were run, and their expression patterns did not differ between untreated and EGCG-treated groups (data not shown). Neuronal and vascular markers (*Tubb3* and *Pecam1*, respectively) seen in Figure 3 were downregulated in all experiment groups (untreated and EGCG-treated), but no differences were depicted between experimental groups *(p* > 0.05). This downregulation of neuronal and vascular markers is commonly observed in established SG molecular atlas databases through gland development [30,31]. Taken together, these findings suggest that EGCG at 7.5 and 15 µg/mL can support the proliferating stem/progenitor niche and the morphogenesis and maturation of pro-acinar buds and the ductal network during regular homeostasis and development.

### 2.2. SG Epithelial Injury Induced by LINAC Radiation

#### 2.2.1. Optimal LINAC Radiation Dose to Induce SG Injury

**Figure 4:**
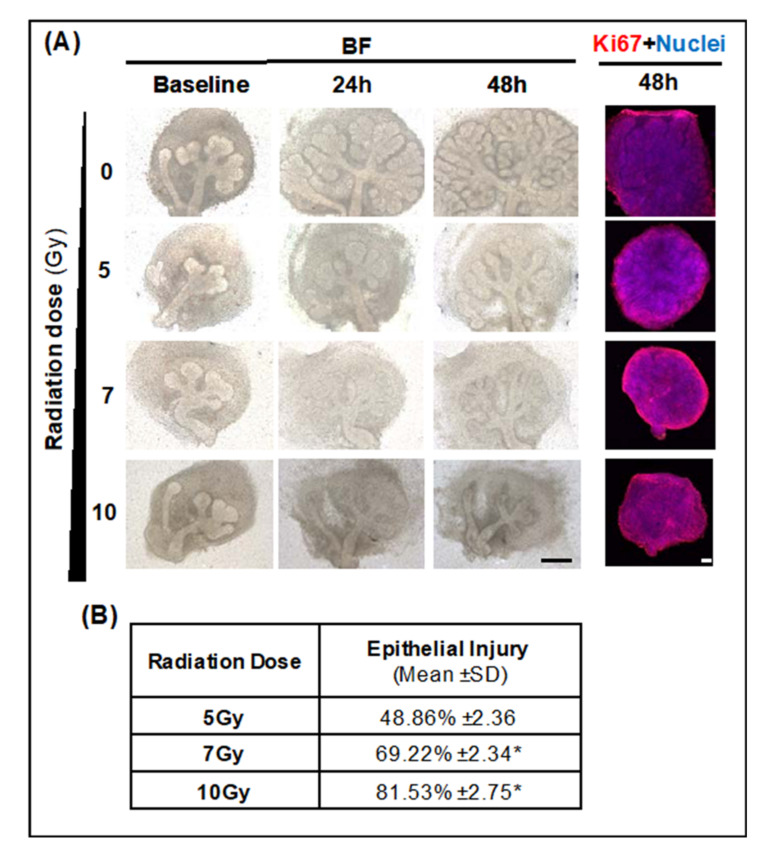
Identifying optimal SG epithelial injury after LINAC radiation exposure with increasing IR doses. (**A**) Bright-field (BF) micrographs and immunofluorescence micrographs of SG stained by whole-mount immunohistochemistry for Ki67 mitotic marker and nuclei. Scale bar: 100 μm. (**B**) Percentage of SG epithelial injury with increasing IR doses based on epithelial growth ratio for each dose and normalized to non-irradiated glands. Data are presented from *n* = 8–11. * *p* < 0.05 when compared to non-irradiated glands using one-way ANOVA with Dunnett’s *post hoc* test.

In Figure 4, a significant epithelial injury (> 50%, *p* < 0.05) could be generated with 7 Gy, and 10 Gy radiation (IR) doses with LINAC. However, a large pool of proliferating Ki67 cells was present at these doses, probably due to low radiation sensitivity as per immunofluorescent images in Figure 4A and as reported in the literature [32]. However, the remaining proliferative epithelia were remarkably low (mean epithelial injury > 80%) with 10 Gy, and thus 7 Gy was selected to investigate EGCG epithelial protection in SG injury models.

#### 2.2.2. EGCG Can Prevent SG Epithelial Damage in a Radiation Injury Model

**Figure 5:**
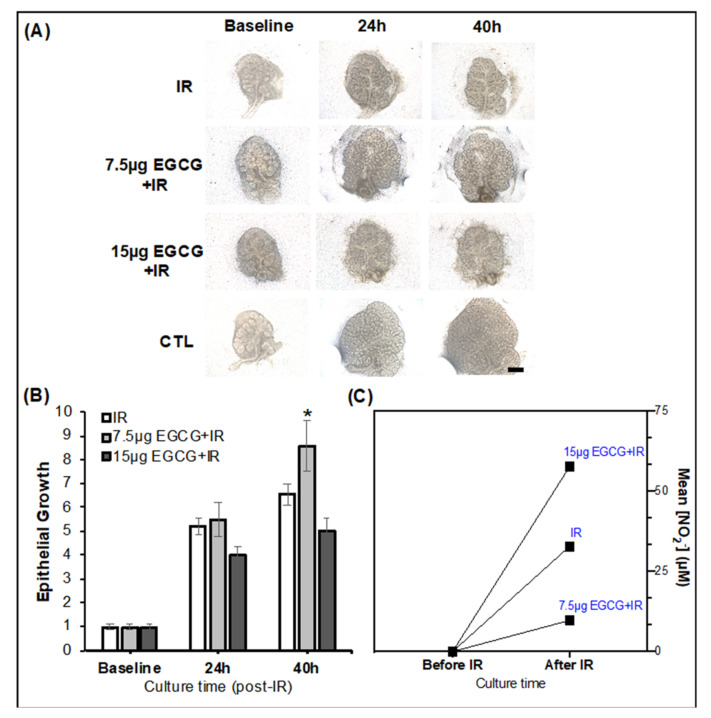
EGCG 7.5 µg/mL increased epithelial growth and decreased oxidative stress markers after IR injury. (**A**) Bright-field micrographs of SG treated with EGCG before IR injury. Mag.: 4×. Scale bar: 100 μm. (**B**) Quantification of epithelial growth ratio during culture with EGCG treatment. Error bars represent SEM from *n* = 12–18. * p < 0.001 when compared to IR using one-way ANOVA with *Dunnett post hoc.* (**C**) Quantification of oxidative stress by determining the levels of nitrites (via a Griess assay) in conditioned media before and after IR and EGCG treatment of the injured SG.

EGCG at 7.5 µg/mL enhanced branching morphogenesis, cleft and secondary duct formation, and epithelial end bud growth after 40 h post-IR as observed in Figure 5A,B when compared to untreated IR controls. Conversely, EGCG at 15 µg/mL could not produce comparable epithelial growth effects and prevent such damage. Moreover, in Figure 5C, media supplementation with EGCG at 7.5 µg/mL decreased the levels of oxidative stress markers (nitrites) in the conditioned media (retrieved from treated glands) in comparison with the media of untreated IR glands. These nitrites are nitric oxide metabolism products and can be promptly measured by a classical Griess assay to assess reactive oxygen species levels. EGCG can exert antioxidant activities in other exocrine glands (e.g., mammary glands), and therefore this effect was expected [26,27]. Thus, herein EGCG at 7.5 µg/mL was found to prevent SG epithelial damage potentially by reducing the oxidative stress generated by IR injury. The pre-treatment of EGCG at 7.5 µg/mL in IR injury models was further investigated to evaluate phenotypic and genotypic changes in the epithelial compartments.

#### 2.2.3. EGCG Prevented Epithelial Damage by Increasing the Progenitor and Pro-Acinar Cell Populations

**Figure 6:**
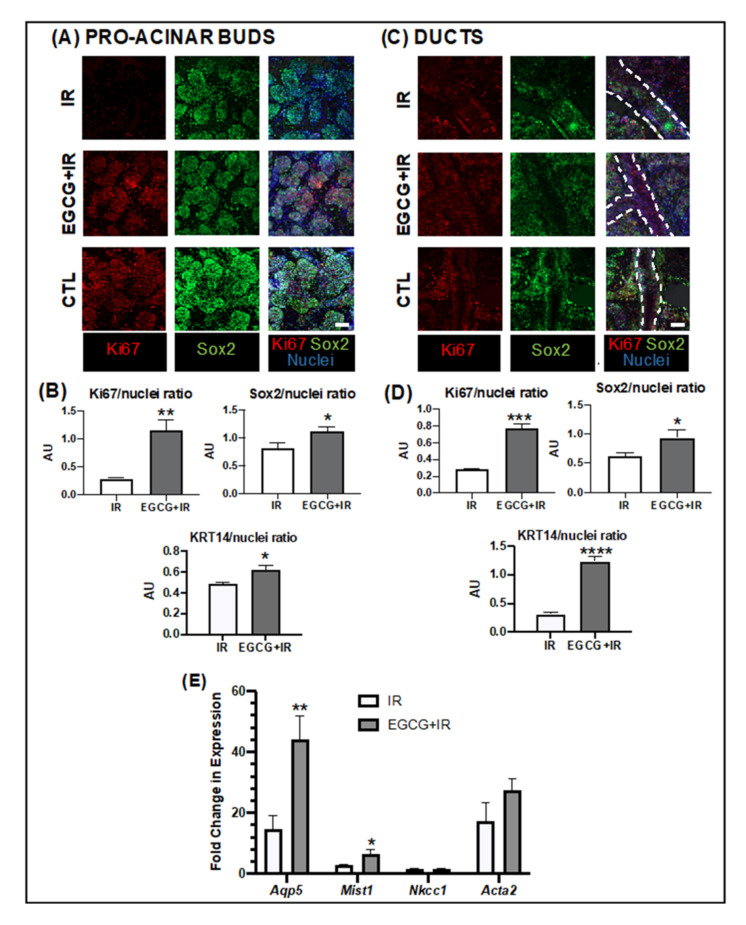
EGCG pre-treatment increased epithelial proliferation and epithelial markers in pro-acinar buds and ducts after IR injury. (**A**,**C**) Micrographs acquired from maximum intensity projections of regions of interest (ROI) in pro-acinar buds (**A**) and ducts (**C**) after whole-mount immunofluorescence and confocal microscopy of untreated glands (IR) and EGCG 7.5 µg/mL pretreated glands. Non-irradiated CTL was used to confirm antibody immuno-reactivity. Mag.: 20×. Scale bar: 50 μm. (**B**,**D**) Graphs are a quantification of Ki67, SOX2, and KRT14 expression based on the immunofluorescence signals at random ROI in pro-acinar buds (**B**) and ducts (**D**) and normalized to total nuclei. Error bars represent SEM from *n* = 5 ROI. Welch’s *Student t-test* were performed between untreated and treated: * *p* < 0.05 (0.0305/0.042), ** *p* < 0.01 (0.0048), *** *p* < 0.001 (0.0006), **** *p* < 0.0001 (**E**) Expression of myoepithelial, acinar and ductal epithelial differentiation markers in the whole gland by qPCR. Data are presented as mean (*n* = 3) of fold change relative to housekeeping gene normalized to baseline. Error bars represent SEM from *n* = 3. *Welch’s Student t-test* were performed between untreated and treated: * *p* < 0.05 (0.045), ** *p* < 0.01 (0.0161).

Gland pre-treatment with EGCG at 7.5 µg/mL increased epithelial proliferation (Ki67^+^ cells) in the pro-acinar buds in Figure 6A,B and ductal compartments in Figure 6C,D. EGCG treatment also enriched stem populations of Sox2^+^ cells in pro-acinar buds in Figure 6A,B and ducts in Figure 6C,D. Progenitor cell populations composed of KRT14^+^ cells were also significantly increased with EGCG in pro-acinar buds in Figure 6B and Figure S1A and ducts in Figure 6D and Figure S1B. Moreover, mature acinar epithelial populations (AQP5^+^ cells) were also upregulated with EGCG treatment as seen in Figure S2 and several acinar epithelial genes like *Aqp5*, *Mist1* (Figure 6E). Myoepithelial genes were also upregulated but not significantly (Figure 6E), but this was not observed with mature ductal genes like *Nkcc1*. Downregulation was seen with other genes, including the ones linked with neuronal and vascular compartments (*Tubb3*, *Pecam1*), though these findings were observed across all experimental groups, including non-irradiated untreated controls (CTL) as displayed in Figure S3. This downregulatory effect was also observed earlier in our study in the homeostasis model in Figure 3.

#### 2.2.4. EGCG Decreased Apoptosis Induced by Radiation Damage

**Figure 7:**
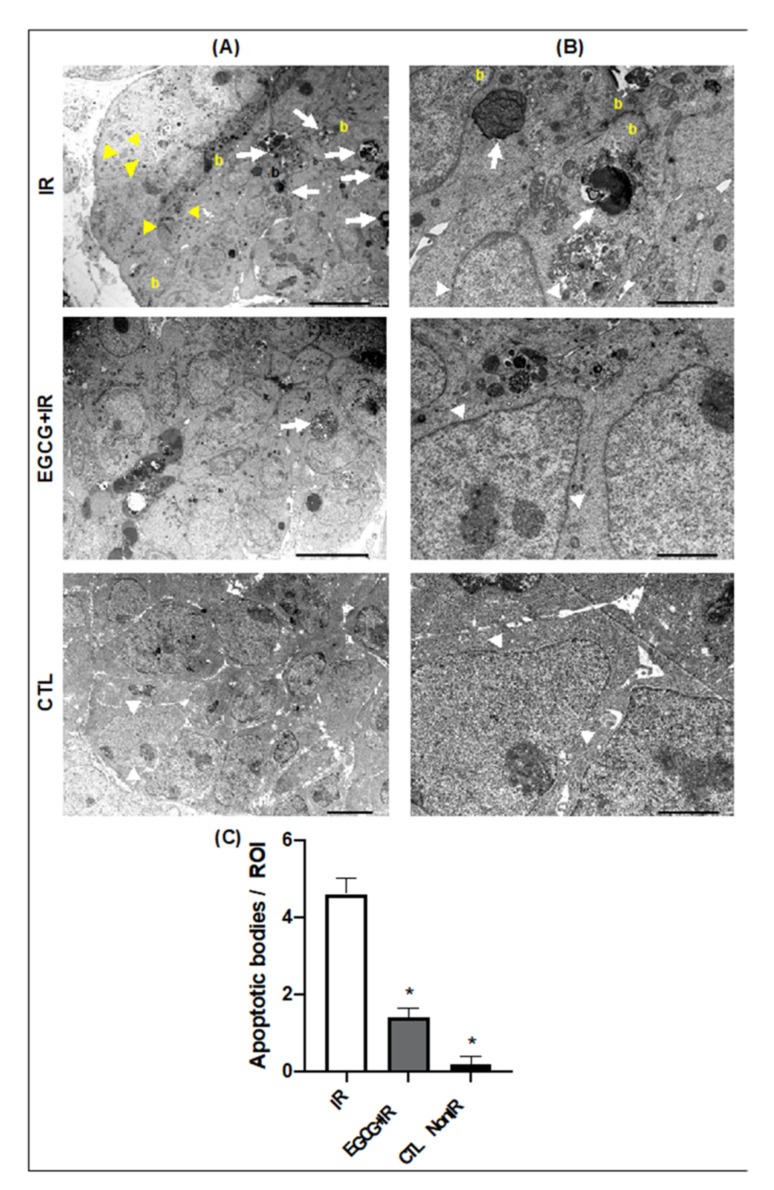
EGCG pre-treatment decreased IR-induced apoptosis and preserved typical nuclear organization. Transmission electron micrographs at low (**A** panel) and high magnifications (**B** panel). Yellow arrowheads show nuclear chromatin fragmentation. White arrows depict apoptotic bodies. White arrowheads indicate the nuclear membrane borders. The yellow letter **b** represents membrane blebbing. (**C**) Apoptotic bodies were counted per region of interest (ROI) electron micrographs taken from in end bud regions with epithelial cells. Error bars represent SEM from *n* = 5. ANOVA with Dunnett post hoc analysis was performed between IR and the other groups: * *p* < 0.0001.

In the cellular ultrastructural analysis, pre-treatment with EGCG decreased the number of apoptotic bodies and preserved the morphologies of the nuclear and plasma membranes in epithelial cells observed in electron micrographs taken from the epithelial bud compartment in Figure 7. These findings were further supported by the limited number of apoptotic bodies (Figure 7C) and decreased expression levels of cleaved Caspase 3 seen in EGCG-treated glands (Figure S4A). Untreated IR glands exhibited a large number of apoptotic bodies (white arrows in Figure 7A,B), membrane blebbing (yellow letter b in Figure 7A,B), and DNA/chromatin breakdown (black arrows in Figure 7A). However, future studies should improve the oral bioavailability of EGCG with the use of nanocarriers.

## 3. Discussion

Firstly, using an SG homeostasis model, we investigated the EGCG concentrations that maintain the growth and maturation of pro-acinar end buds and the formation of secondary ducts. In this model, EGCG at 7.5–15 µg/mL supported the regular epithelial branching morphogenesis events leading to an increase in end-bud epithelial growth, epithelial cleft formation, and secondary duct development. These findings were confirmed at genotypic and phenotypic levels. Furthermore, there was an enrichment of acinar epithelial markers through culture and ductal markers but to a lesser extent on both EGCG-treated and untreated normal glands. Similarly, Yamamoto and colleagues’ early reports [28] showed that EGCG 5.75–23 µg/mL maintained cell viability at about 105–108% in in vitro cultures of immortalized SG acinar cells when compared to untreated glands. However, in the same study, EGCG at 23 µg/mL significantly reduced immortalized ductal cells’ viability to 87%. Thus, the importance of evaluating the biological effects of EGCG in the developing SG epithelial organ is paramount since it can have antagonistic effects on the proliferation and potentially on the maturation of different epithelial cells (stem cells, acinar, ductal, myoepithelial).

Regarding the SG IR injury model, glands pretreated with EGCG at 7.5 µg/mL promoted epithelial proliferation in both the pro-acinar buds and the ductal network. These effects may be the consequence of the lower oxidative stress levels observed after IR with this EGCG concentration. This is in part supported by previous literature in auto-immune SG injury models where submandibular glands of mice fed with 0.2% EGCG in water have a remarkably decreased expression of antioxidant enzymes relative to plain water-fed mice [21]. This antioxidant effect of EGCG has been reported in other glands and epithelial tissues [27,33]. However, the therapeutic window towards EGCG cytoprotection may be limited since slightly higher concentrations of EGCG (23 µg/mL and above) have more recently been shown to reduce the viability and alter the cell cycle of immortalized epithelial cells [34]. Future studies shall evaluate the potential for nanocarriers to increase the oral bioavailability of EGCG.

Moreover, EGCG also enriched pro-acinar buds and ducts with stem and progenitor cell populations (Sox2^+^ and KRT14^+^) and with more mature pro-acinar epithelial cells (AQP5^+^) after IR injury. Several acinar epithelial and myoepithelial genes were also upregulated; however, this was not observed with ductal epithelial genes (*Krt5*, *Krt19*, *Nkcc1*). These findings may explain why green tea formulas rich in EGCG were able to partially restore salivary function in patients with radiotherapy-induced xerostomia and improve saliva secretion [35]. In this latter phase II clinical study, EGCG may be promoting the proliferation and maturation of the remaining pro-acinar stem/progenitor cells after radiation damage, according to our gland phenotypic and genotypic observations herein (Figure 6). Downregulation of markers linked with neuronal and vascular compartments (*Tubb3*, *Pecam1*) was observed in EGCG-treated and non-IR glands as expected and similar to the expression patterns seen in our homeostasis model.

Epithelial apoptosis phenomena are commonly observed after radiation injury to the SG [13,36], and thus these should also be assessed after EGCG treatment. In this study, when glands were pretreated with EGCG, apoptotic biomarkers such as Caspase 3 and apoptotic bodies declined, and most epithelial cells retained their normal membrane and nuclear morphology (Figure 7). Radioiodine therapy (RIT) is also known to induce oxidative stress leading to SG epithelial apoptosis and hypofunction. Thus recently, investigators have found that EGCG administered before RIT in mice could reduce the number of apoptotic cells and protect from RIT-induced SG damage in a way comparable to amifostine, a FDA-approved antioxidant agent [37].

The systemic absorption and oral bioavailability of EGCG can be remarkably reduced due to its poor intestinal permeability and stability [38]. To tackle this limitation, recent studies have used either chitosan nanoparticles or folic acid-functionalized nanostructured lipid carriers to enhance the EGCG intestinal absorption and oral bioavailability [38,39]. Thus, our future studies will use novel nanocarriers to improve the oral bioavailability of EGCG towards the protection of SG secretory epithelia from radiation injury. One of such nanocarriers that we are exploring is hydrogel-based nanofiber mats that can promote agents’ local delivery to the salivary glands [40]. Hence, local delivery of EGCG would promote a more effective oral bioavailability of this agent and confer protection against IR-induced apoptosis and enrich the pro-acinar epithelial cell populations leading towards an improved acinar salivary secretion.

## 4. Materials and Methods

### 4.1. Salivary Gland Ex Vivo Organ Culture

All animal procedures were approved by the Institutional Animal Care and Use Committee (IACUC) at the Chulalongkorn University Laboratory Animal Center under the protocol number 1973004. The study was conducted according to the guidelines of the Declaration of Helsinki, and approved by the Institutional Biosafety Committee of the Chulalongkorn University Faculty of Dentistry (DENT CU-IBC 006/2019 on March 2019 and DENT CU-IBC 006/2020 on March 2020). Fetal submandibular glands were surgically dissected from ICR mouse embryos at embryonic day E13 under a stereomicroscope (SZH10 model from Olympus, Tokyo, Japan or SMZ1270i model from Nikon Corporation, Tokyo, Japan), as previously described [13,40]. All reagents and plasticware were purchased from Thermo Fisher Scientific (Waltham, MA, USA) or Sigma-Aldrich (Merck, St. Louis, MO, USA) and its subsidiaries unless stated otherwise. After dissections, glands were cultured on porous polycarbonate membranes (Whatman^TM^ Nucleopore) and placed at the air/medium interface at the center well of 50 mm dishes as previously described [13,40]. These membranes were kept floating on growth media (GM) composed of phenol-free DMEM/F12, 1% penicillin/streptomycin, 150 µg/mL ascorbic acid and 100 µg/mL human holo-transferrin. GM was then replaced at baseline by fresh GM supplemented with EGCG (E4143-50M6, Sigma-Aldrich) from concentrations ranging from 7.5 to 30 µg/mL, and glands were kept inside an incubator with 5% CO_2_ and at 37 °C for up to 72 h. Positive controls (CTL) had GM only (without EGCG supplementation). Every day, 50% of the culture media was replaced by fresh GM.

To set up the radiation injury models, salivary glands were treated with EGCG at baseline and then underwent 5–10 Gy irradiation at 24 h of culture using a 6 MV Varian TrueBeam^TM^ LINAC radiotherapy system (Varian Medical Systems, Palo Alto, CA, USA). After such a procedure, media was replaced as described above. Both the homeostasis and injury models are displayed in Figure 1.

### 4.2. Quantification of SG Epithelial Growth

Salivary glands were observed under bright-field and phase-contrast microscopy at baseline, 24, 40, 48 and 72 h, and images were acquired at 5–10× magnification with a Leica DMi1 light microscope (Leica Microsystems, Wetzlar, Germany). The epithelial growth during the SG branching morphogenesis process was determined by counting the number of epithelial buds at every time point on a blinded approach using ImageJ (NIH, Bethesda, MD, USA) as per our previous studies [13,40]. Epithelial growth ratio was quantified at each time point by dividing the end bud numbers at each time point with the end bud number at baseline. Each experiment had 4–5 glands per experimental group and was run at least three independent times.

### 4.3. Whole-Mount Immunohistochemistry

Untreated and treated SG with EGCG at 7.5 µg/mL (optimized concentration) were fixed after 72 h of culture using 4% paraformaldehyde (PFA) for 20 min at room temperature (RT). The immunohistochemistry protocol has been published elsewhere [40,41]. Briefly, SMGs were permeabilized with 0.1% Triton X for 15 min and washed using 1× phosphate-buffered saline (PBS) three times. Tissues were blocked overnight with 10% horse serum, 5% bovine serum albumin (BSA), and 1.8% mouse-on-mouse IgG blocking reagent (M.O.M kit, Vector laboratories, Burlingame, CA, USA) in 0.1% PBS-Tween 20. SMGs were incubated with primary antibodies overnight at 4 °C according to each antibody optimal dilution as described in Table S1. A solution containing acetone and methanol (1:1 dilution) was used to add further specific primary antibodies not compatible with PFA. Next, glands were washed thrice in PBS and incubated with secondary antibodies according to the dilutions in Table S1. Nuclei were then labeled with Hoechst 33342 (1:1000 dilution, catalog number R37605, Invitrogen, Thermo Fisher Scientific, Waltham, MA, USA). Next, glands were mounted on a glass side with spacers and filled with resin-mounted media (Electron Microscopy Sciences, Hatfield, PA, USA). Fluorescence image acquisition of fluorescently-labeled proteins and nuclei was performed using laser scanning confocal microscopes (Zeiss LSM700 and LSM800, Jena, Germany) z-stacking (with 3 μm intervals between each scan) and maximum intensity projections were generated at low and high magnifications. Quantification of fluorescence intensity was performed using ImageJ software using at least five random regions of interest, and data were normalized to total nuclear fluorescence.

### 4.4. Oxidative Stress Measurement

A classical Griess assay kit (G2930, Promega, Madison, WI, USA) was used to measure nitrite levels. Briefly, 50 μL of conditioned medium from baseline and post-radiation untreated and EGCG-treated glands were added into 96-well flat bottom plate and mixed with 50 μL sulfanilamide solution and incubated for 5–10 min at RT inside a dark chamber. Next, 50 μL of N-1-napthylethylenediamine dihydrochloride (NED) solution was subsequently added, followed by an incubation step of 10 min. Nitrite standard solutions were used to produce a standard curve. All media was kept phenol-free, and wells with fresh GM only were used to subtract the background levels of nitrites in the fresh media. The absorbance at 490 nm was measured every 30 min using a GloMax^®^ Discover Microplate Reader (Promega) until saturation levels were reached in the maximum nitrite standard solutions.

### 4.5. Gene Expression Arrays

Untreated and treated SG with EGCG at baseline (fresh gland state) and at 72 h of culture were placed into RNA lysis buffer, and RNA was extracted, isolated, and purified using Monarch^®^ Total RNA Miniprep Kit (T2020G, New England Biolabs, Ipswich, MA, USA), according to the manufacturer’s protocol. Next, reverse transcriptase enzyme SuperScript™ III First-Strand Synthesis System (Invitrogen, Thermo Fisher Scientific) was used to synthesized cDNA from total RNA, and cDNA was diluted to 1 ng/μL in nuclease-free water. Next, 1 ng of cDNA was used to perform SYBR^®^ green-based quantitative polymerase chain reaction (qPCR). All oligonucleotide primers (forward and reverse sequences) are listed in Table S2. Each qPCR reaction was performed with 10 μL cDNA, 9.5 μL QuantiTect SYBR^®^ Green PCR kit (QIAGEN, Hilden, Germany), 0.5 μL of forward and reverse oligonucleotide primer mix and run on an Applied Biosystems QuantStudio 3 Real-Time PCR System (Applied Biosystems, Thermo Fisher Scientific) and Data Assist Software v3.01. Data were evaluated using 2^−(ddCT)^ method to quantify relative expression of target genes at 72 h upon normalization with a reference housekeeping gene (*Rsp29)* and relative to the baseline levels [13,42].

### 4.6. Transmission Electron Microscopy

For cellular ultrastructural analysis, glands at 72 h of culture were fixed with 3% glutaraldehyde in 0.1 M phosphate buffer and kept in the fridge until processing [43]. Tissues were then rinsed in 0.1 M phosphate buffer three times. Post-fixation was done in 2% osmium tetroxide in the same buffer solution at 4 °C for 45 min, and tissues were dehydrated in a graded series of alcohol and embedded in Spurr’s resin:propylene oxide (1:1) for 10 min, Spurr’s resin:propylene oxide (3:1) for 15 min, and 100% Spurr’s resin for 15 min three times. The embedding process continued for 16 h at 70 °C. Semi-thin sections were obtained using glass knives with Ultracut E Microtome (Leica Microsystems, Wetzlar, Germany), and ultra-fine sections (90–100 nm) were mounted on copper grids of 100 meshes. The grids were stained by uranyl acetate and lead then observed in a JEM-1400 transmission electron microscopy (JEOL, Peabody, MA, USA) adjusted to 200 kV. Control glands (non-irradiated) were used as positive controls. According to random regions of interest in electron micrographs from end bud regions containing ten epithelial cells. Apoptotic bodies were counted using ImageJ software.

### 4.7. Statistical Analysis

All data are displayed as mean ± standard error of the mean and were normally distributed. Sample sizes are displayed under the caption of each graph. For a two-group comparison, multiple unpaired *Welch’s Student t*-test was performed. For more than two group-comparisons, one-way ANOVA with Tukey or Dunnett’s *post hoc* analysis was run. The significance level was set at *p* < 0.05. All statistical tests were performed using GraphPad Prism version 9 (GraphPad Software, San Diego, CA, USA).

## Data Availability

The data presented in this study are available on request from the corresponding author. The data are not publicly available due to intellectual property restrictions.

## Supplementary Materials

The following are available online at https://www.mdpi.com/1422-0067/22/6/3162/s1, Table S1: List of primary and secondary antibodies were used. Manufacturers: Life Technologies and Invitrogen are subsidiaries of Thermo Fisher Scientific (USA), Santa Cruz (USA), Abcam (UK), Cell Signaling Technologies (USA), and R&D systems (USA); Table S2: List of oligonucleotide primer sequences; Figure S1: Expression of epithelial progenitors in EGCG-treated glands after IR injury. Expression of cytokeratin 14 (KRT14) progenitor markers in pro-acinar endbud compartments (A) and in ductal compartments (B) after immunofluorescence staining. Images shown are maximum intensity projections. Mag.: 20×. Scale bar: 100 μm; Figure S2: Expression of differentiated acinar epithelial and mitotic markers in EGCG pretreated glands after IR injury. SG were immuno-stained with Aquaporin 5 (AQP5), a mitotic marker (Ki67) and incubated with a nuclear stain. Mag.: 10×. Scale bar: 200 μm; Figure S3: Heatmap with mean expression of other stem/progenitor, ductal epithelial, neuronal and vascular markers in the whole gland by qPCR. Data are presented as mean (*n* = 3) of fold change relative to housekeeping gene normalized to baseline from *n* = 3. *Welch’s Student t-test* were performed between untreated and treated but not significant difference was observed. CTL represent non-irradiated untreated controls; Figure S4: Expression of pro-apoptotic Caspase 3 marker in EGCG pretreated glands after IR injury. SG were immuno-stained with cleaved-caspase 3 (CASP3), Ꞵ-3 tubulin (TUBB3) to depict the boundaries of acinar buds where terminal neurons synapse. SG were also incubated with a nuclear stain. Mag.: 40×. Scale bar: 100 μm.
