# Supplementary material for: Epigallocatechin-3-Gallate Protects Pro-Acinar Epithelia Against Salivary Gland Radiation Injury
Source: Int J Mol Sci. 2021 Mar 19;22(6):3162. doi: 10.3390/ijms22063162 (PMC8003734; doi:10.3390/ijms22063162)
Supplement: Supplementary file 1 [file ijms-22-03162-s001.pdf]

Supplementary Data: Figures and Tables

Sulistiyani et al. IJMS Mar 2021

Revised 17 March 2021

Table S1. List of primary and secondary antibodies were used. Manufacturers: Life Technologies and Invitrogen are subsidiaries of Thermo Fisher Scientific (USA), Santa Cruz (USA), Abcam (UK), Cell Signaling Technologies (USA), and R&D systems (USA).

	Antibody	Dilution	Manufacturer / Catalog number
**Secondary Antibodies**	Alexa Fluor 488	1:200	Life Technologies / A21200
	Alexa Fluor 594	1:200	Life Technologies / A21442
	Alexa Fluor 594	1:200	Life Technologies / A21442
	Alexa Fluor 488	1:200	Life Technologies / A11055
**Primary Antibodies**	Sox2	1:100	Santa Cruz / SC17320
	Ki67	1:200	Invitrogen / PA5-19462
	KRT14	1:500	Abcam / 181595
	AQP5	1:100	Abcam / AB92320
	Cleaved Caspase 3	1:200	Cell Signaling Technologies / 9664S
	β-3 tubulin (Tuj-1)	1:100	R&D systems / MAB1195

Table S2. List of oligonucleotide primer sequences

Gene	Forward sequence	Reverse sequence
Ki67	CATACCTGAGCCCATCACCA	GCTTTGCTGCATTCCGAGTA
Sox 2	CAGCATGTCCTACTCGCAGCAG	TGGAGTGGGAGGAAGAGGTAACC
Sox10	ATCAGCCACGAGGTAATGTCCAAC	ACTGCCCAGCCCGTAGCC
Krt14	CAGCCCCTACTTCAAGACCA	GTCGATCTGCAGGAGGACAT
Aqp5	TCTACTTCTACTTGCTTTTCCCCTCCTC	CGATGGTCTTCTTCCGCTCCTCTC
Krt5	TCCTGTTGAACGCCGCTGAC	CGGAAGGACACACTGGACTGG
Acta2	GGAGAAGCCCAGCCAGTCGC	AGCCGGCCTTACAGAGCCCA
Krt19	CCTCCCGAGATTACAACCACT	GGCGAGCATTGTCAATCTGT
Mist1	GCTGACCGCCACCATACTTAC	TGTGTAGAGTAGCGTTGCAGG
Nkcc1	TTCCGCGTGAACTTCGTGG	TTGGTGTGGGTGTCATAGTAGT
Pecam1	TCCAACAGAGCCAGCAGTATGAGG	TCCAATGACAACCACCGCAATGAG
Tubb3	CCAGAGCCATCTAGCTACTGACACTG	AGAGCCAAGTGGACTCACATGGAG
Rsp29	GGAGTCACCCACGGAAGTTCGG	GGAAGCACTGGCGGCACATG

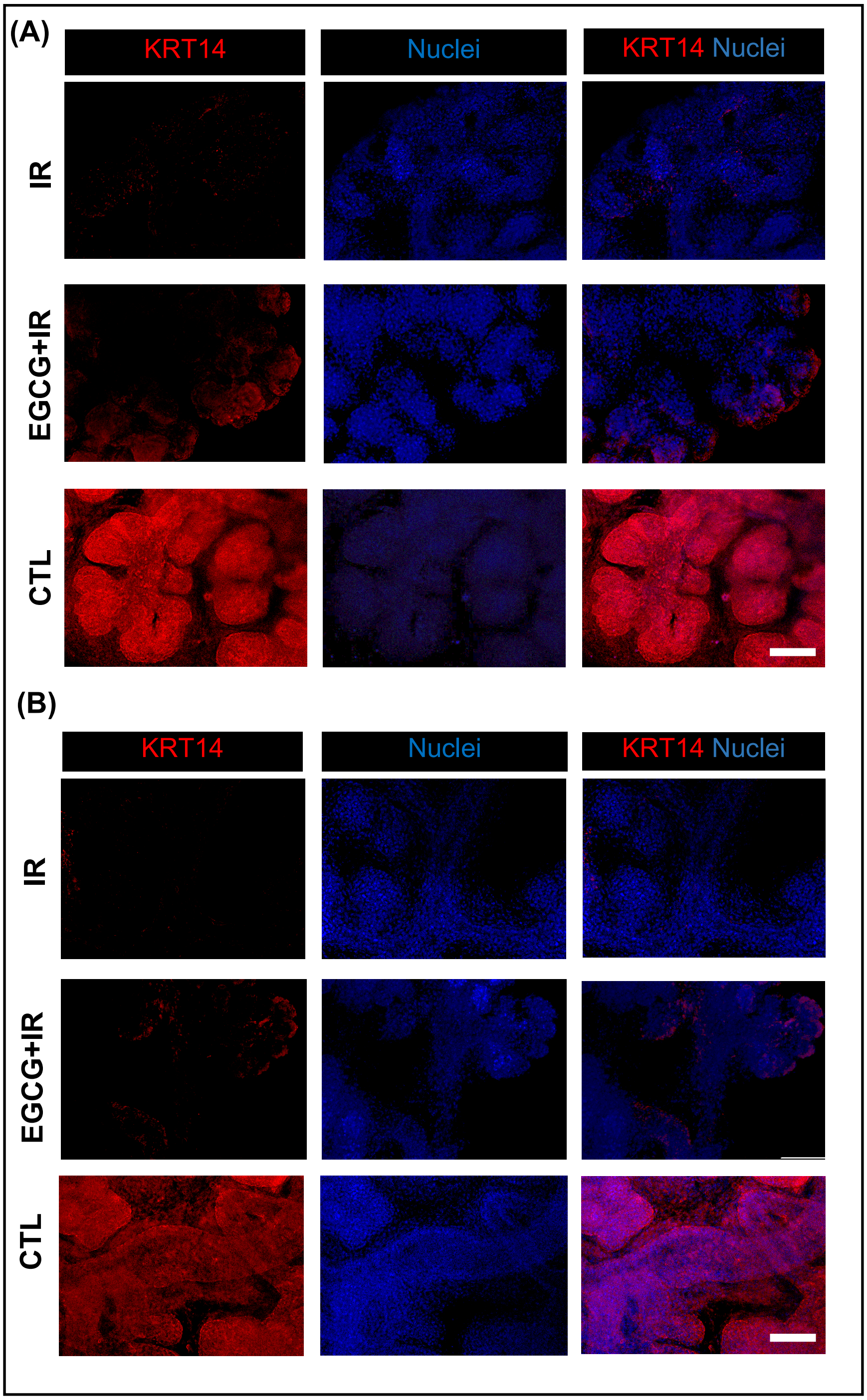

Figure S1. Expression of epithelial progenitors in EGCG-treated glands after IR injury. Expression of cytokeratin 14 (KRT14) progenitor markers in pro-acinar endbud compartments (**A**) and in ductal compartments (**B**) after immunofluorescence staining. Images shown are maximum intensity projections. Mag.: 20X. Scale bar: 100µm.

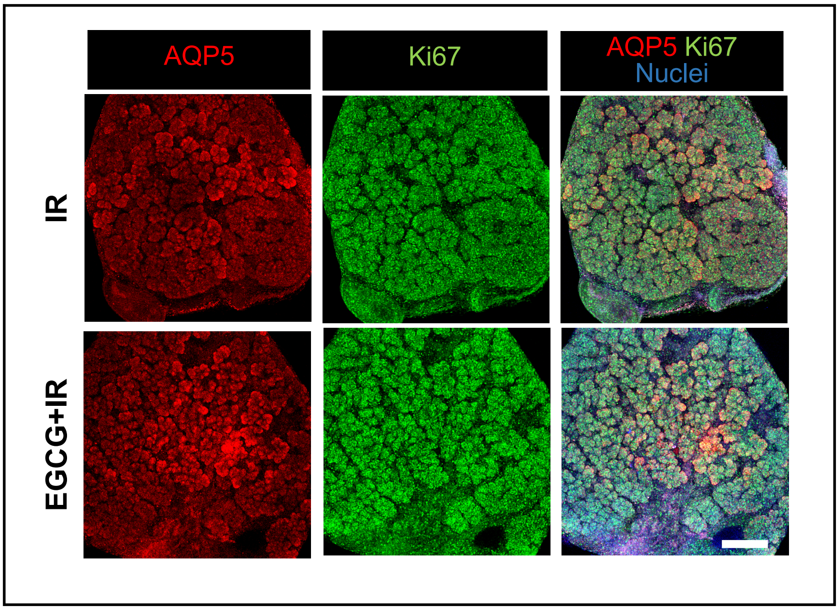

Figure S2. Expression of differentiated acinar epithelial and mitotic markers in EGCG pre-treated glands after IR injury. SG were immuno-stained with Aquaporin 5 (AQP5), a mitotic marker (Ki67) and incubated with a nuclear stain. Mag.: 10X. Scale bar: 200µm.

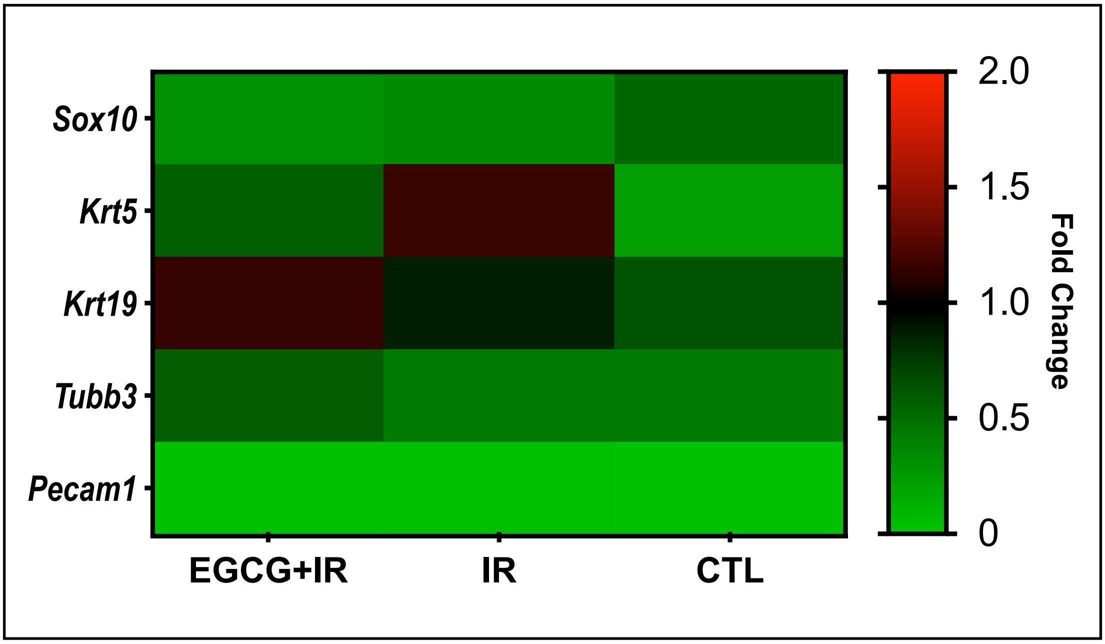

Figure S3. Heatmap with mean expression of other stem/progenitor, ductal epithelial, neuronal and vascular markers in the whole gland by qPCR. Data are presented as mean (n = 3) of fold change relative to house keeping gene normalized to baseline from n = 3. *Welch's Student t-test* were performed between untreated and treated but not significant difference was observed. CTL represent non-irradiated untreated controls.

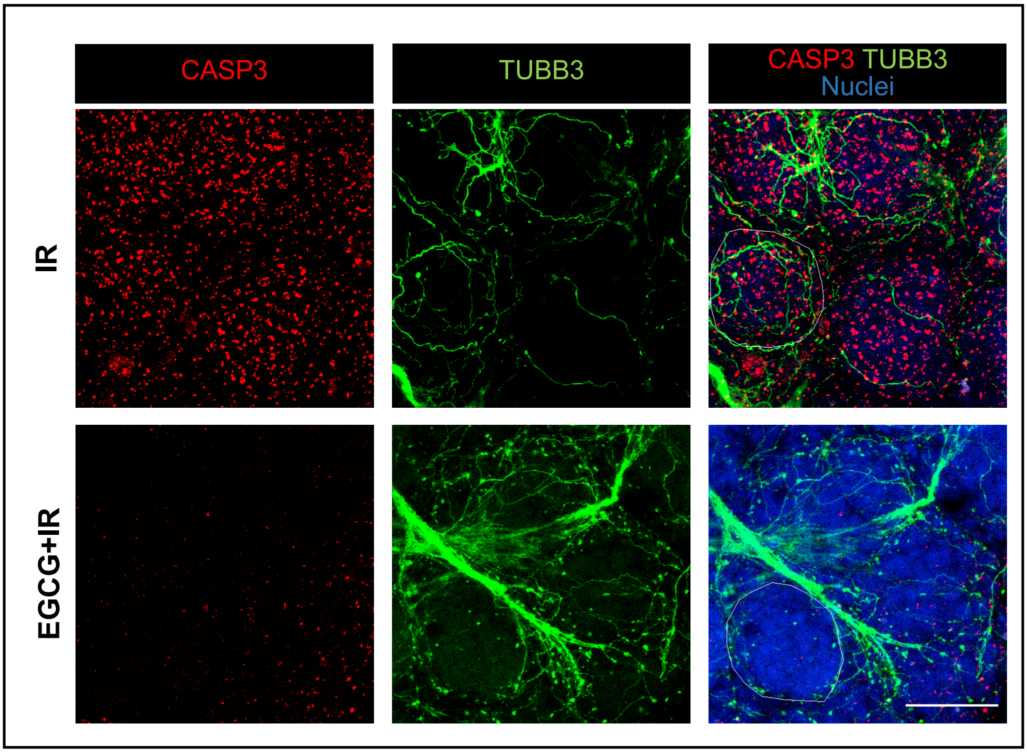

Figure S4. Expression of pro-apoptotic Caspase 3 marker in EGCG pre-treated glands after IR injury. SG were immuno-stained with cleaved-caspase 3 (CASP3), β-3 tubulin (TUBB3) to depict the boundaries of acinar buds where terminal neurons synapse. SG were also incubated with a nuclear stain. Mag.: 40X. Scale bar: 100μm.
